# Tuberculosis infection in children visiting friends and relatives in countries with high incidence of tuberculosis: A study protocol

**DOI:** 10.1097/MD.0000000000022015

**Published:** 2020-09-04

**Authors:** Antoni Soriano-Arandes, Joan A. Caylà, Alessandra Queiroga Gonçalves, Àngels Orcau, Antoni Noguera-Julian, Emma Padilla, Elisabet Solà-Segura, Neus Rius Gordillo, María Espiau, Mónica G. García-Lerín, Maria Àngels Rifà-Pujol, Jordi Gómez i Prat, Esperança Macia-Rieradevall, Andrea Martin-Nalda, Maria Eril-Rius, José Santos Santiago, Lídia Busquets-Poblet, Raisa Morales Martínez, Tomàs Maria Pérez-Porcuna

**Affiliations:** a Unitat de Patologia Infecciosa i Immunodeficiències Pediàtriques, Vall d’Hebron Institut de Recerca (VHIR), Hospital Universitari Vall d’Hebron; b Fundació de la Unitat d’Investigació en Tuberculosi de Barcelona, Barcelona; c Unitat de Suport a la Recerca Terres de l’Ebre, Fundació Institut Universitari per a la Recerca a l’Atenció Primària de Salut Jordi Gol i Gurina (IDIAPJGol); d Unitat Docent de Medicina de Familia i Comunitària, Tortosa-Terres de l’Ebre, Institut Català de la Salut, Tortosa, Tarragona; e Servei d’epidemiologia, Agència de Salut Pública de Barcelona, Barcelona; f Centro de Investigación Biomédica en Red de Epidemiología y Salud Pública (CIBERESP), Madrid; g Malalties Infeccioses i Resposta Inflamatòria Sistèmica en Pediatria, Unitat d’Infeccions, Servei de Pediatria, Institut de Recerca Pediàtrica, Hospital Sant Joan de Déu, Barcelona; h Departament de Pediatria, Universitat de Barcelona, Barcelona; i Red de Investigación Translacional en Infectología Pediátrica (RITIP), Madrid; j Àrea de Microbiologia de Catlab, Terrassa; k Equip d’Atenció Primària Vic Nord, Institut Català de la Salut, Vic; l Servei de Pediatria Hospital Universitari Sant Joan de Reus, Reus; m Atenció Primària, Fundació Assistencial Mútua Terrassa, Terrassa; n Equip d’Atenció Primària Tona, Institut Català de la Salut, Tona; o Equip de Salut Pública i Comunitària de la Unitat de Salut Internacional Drassanes-Hospital Universitari Vall d’Hebron, Servei de Medicina Preventiva de Vall d’Hebron, Barcelona; p Equip d’atenció primària Manlleu, Institut Català de la Salut, Manlleu; q Grup de recerca infecció en el pacient pediàtric immunodeprimit, Vall d’Hebron Institut de Recerca (VHIR), Hospital Universitari Vall d’Hebron, Barcelona; r Centre de Diagnòstic i Investigació per a Immunodeficiències Primàries Jeffrey Modell, Barcelona; s Equip d’atenció primària La Vall del Ges, Institut Català de la Salut, Torelló; t Centre de Salut Internacional i Malalties Transmissibles Drassanes/Vall d’Hebron. Programa de Salut Internacional de l’ICS (PROSICS), Barcelona; u Servei de pediatría, CAP El Remei, L’Equip d’Assistència Primària de Vic, Vic; v Unitat clínica de Tuberculosi i Salut Internacional, Fundació de Docència i Recerca Mútua Terrassa, Servei de Pediatria, Hospital Universitari Mútua Terrassa, Terrassa, Spain.

**Keywords:** children, incidence, interferon-gamma release assay, latent tuberculosis, travel-related infection, tuberculin test, tuberculosis

## Abstract

**Introduction:**

Tuberculosis (TB) is a global infectious disease. In low-incidence countries, paediatric TB affects mostly immigrant children and children of immigrants. We hypothesize that these children are at risk of exposure to *Mycobacterium tuberculosis* when they travel to the country of origin of their parents to visit friends and relatives (VFR). In this study, we aim to estimate the incidence rate and risk factors associated to latent tuberculosis infection (LTBI) and TB in VFR children.

**Methods and analysis:**

A prospective study will be carried out in collaboration with 21 primary health care centres (PCC) and 5 hospitals in Catalonia, Spain. The study participants are children under 15 years of age, either immigrant themselves or born to immigrant parents, who travel to countries with high incidence of TB (≥ 40 cases/100,000 inhabitants). A sample size of 492 children was calculated. Participants will be recruited before traveling, either during a visit to a travel clinic or to their PCC, where a questionnaire including sociodemographic, epidemiological and clinical data will be completed, and a tuberculin skin test (TST) will be performed and read after 48 to 72 hours; patients with a positive TST at baseline will be excluded. A visit will be scheduled eight to twelve-weeks after their return to perform a TST and a QuantiFERON-TB Gold Plus test. The incidence rate of LTBI will be estimated per individual/month and person/year per country visited, and also by age-group.

**Ethics and dissemination:**

The study protocol was approved by the Clinical Research Ethics Committee of the Hospital Universitari Mútua Terrassa (code 02/16) and the Clinical Research Ethics Committee of the Fundació Institut Universitari per a la Recerca a l’Atenció Primària de Salut Jordi Gol i Gurina (code P16/094). Articles will be published in indexed scientific journals.

**Trial registration:**

Clinical-Trials.gov: NCT04236765

## 1 Introduction

It is estimated that one third of the world population is infected with *Mycobacterium tuberculosis* and that 5% to 10% of those infected will develop tuberculosis (TB) during their lifetime.^[1]^ Current global figures indicate that 10 million people develop TB every year, of which 1.1 million are children.^[2]^

It is important to underscore that children have a greater risk of developing active TB after primary infection, especially before their fifth birthday.^[3]^ Significantly, children infected with *M tuberculosis* that do not develop the disease and that do not receive preventive treatment will constitute a reservoir of TB in the future.^[4]^ Consequently, the identification and administration of preventive treatment to children with latent tuberculosis infection (LTBI) are critical to the global TB control and eradication efforts.^[5–7]^

In countries with low TB incidence, the burden of disease is notably higher in the immigrant than in the autochthonous population.^[8–10]^ Importantly, this higher incidence of tuberculosis persists beyond the first years after arrival to the host country.^[11,12]^ In fact, most new paediatric TB cases in western countries occur in immigrant children and autochthonous children born to immigrant parents.^[5,8,9]^

In Europe, TB and hepatitis C are the main imported infections among the immigrant population.^[13]^ The recent increase in international travel of immigrants to their countries of origin to visit friends and relatives (VFR)^[14–16]^ and subsequent exposure to *M tuberculosis* in countries with incidences of TB higher than in the host countries could partially explain the differences in incidence between children of immigrant families and children from the autochthonous population.^[17]^ It seems plausible to associate travel-related factors with risk of contagion, including the intensity of transmission (proportional to the local TB incidence), the relationship and contact with the local population, the duration of the stay, and individual host factors.^[18]^

However, the risk of LTBI is difficult to estimate, firstly because of the lack of a gold standard for the diagnosis, and secondly due to the long incubation period before the development of the TB disease, specially in people over 5 years of age.^[18]^

Due to the little evidence on the risk factors associated to LTBI and TB in VFR children, it is crucial to elucidate the epidemiology of TB in the immigrant population and their children to better inform public health policies aimed at reducing the number of TB cases.^[19]^

The objectives of this study are (1) to estimate the incidence rate of LTBI and TB in paediatric VFR travellers when returning from the countries of origin of their parents and (2) to identify risk factors associated with LTBI and TB.

## 2 Methods and design

An interventional, single group assignment, multicentre study will be carried out from June 2017 to December 2020 in Catalonia. The incidence rate of LTBI/TB in Catalonia is 12.6 cases/100,000 inhabitants and 36.3 cases/100,000 inhabitants among the general and the immigrant population, respectively.^[20]^ BCG vaccination is not included in the national programme of immunization.

### 2.1 Setting and participants

Participants of the study are children that meet the inclusion criteria, attended at any of the 5 pre-travel health clinics or at any of the 21 primary health centres (PCC) involved in the study.

#### 2.1.1 Inclusion criteria (all criteria must be met)

**Figure 1:**
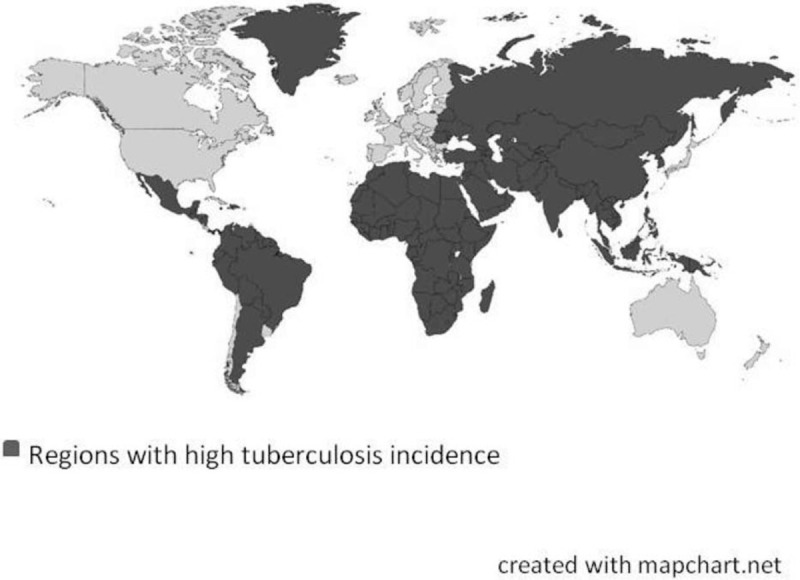
Countries considered in the study for having regions with an incidence of tuberculosis of 40 cases/100,000 inhabitants or greater.

- Children less than 15 years of age, either immigrants or children born in Spain to immigrant parents.
- Children with at least one of the parents from a high TB incidence country. We define “high TB incidence country” as a country with a TB incidence rate 3 times higher than in Catalonia, namely 40 cases/100,000 inhabitants or greater.^[6,20]^ Countries with official reports describing an incidence less than the value proposed but with some regions with ≥40 cases /100,000 inhabitants will also be considered of high incidence (Fig. 1 and Table 1 ).
- Children travelling and accompanying at least one of the parents to their country of origin.
- The duration of the trip must last a minimum of 21 days.
- Informed consent must be obtained from the parents or legal guardian.

**Table 1:**
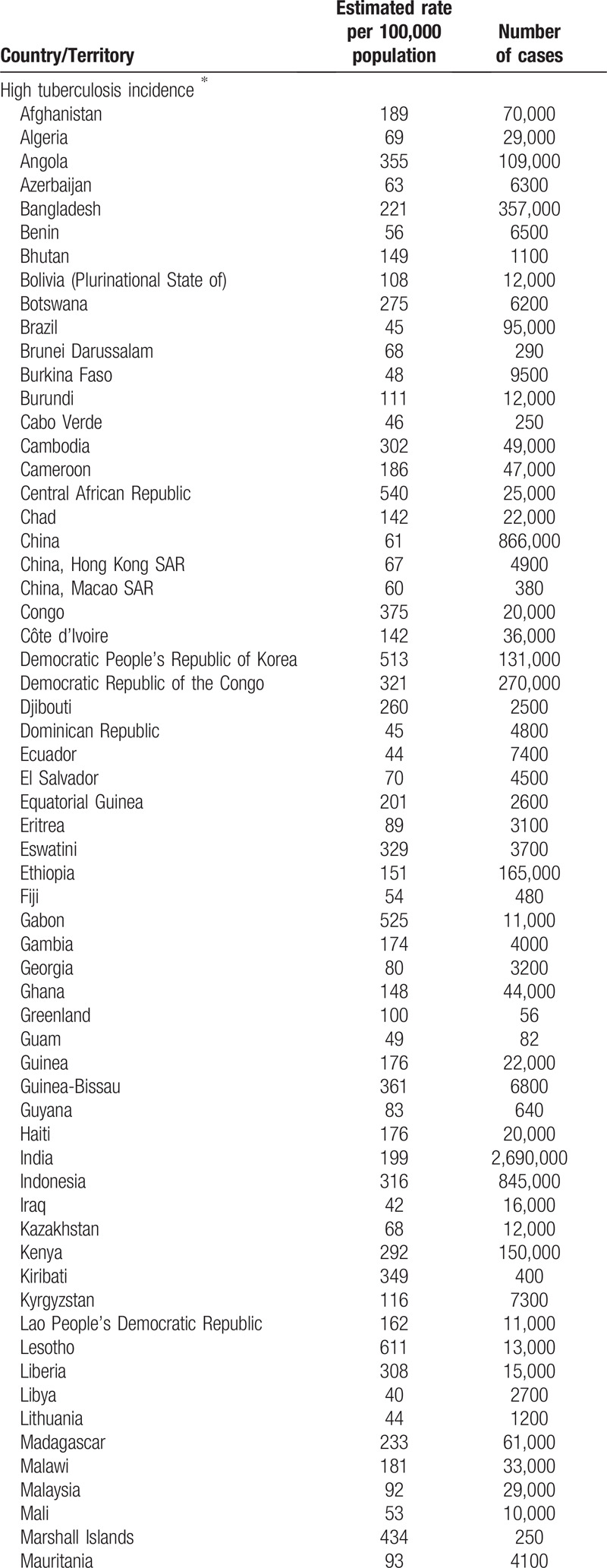
High and low tuberculosis incidence countries.

**Table 1 (Continued):**
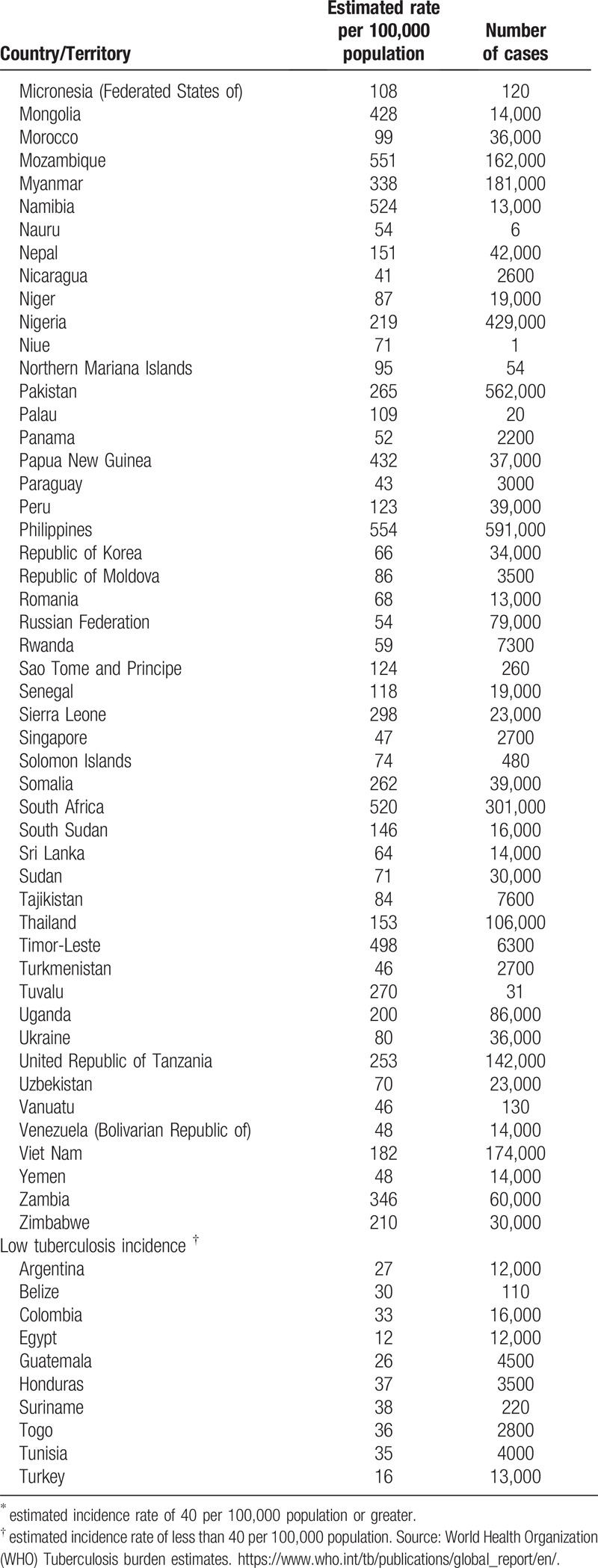
High and low tuberculosis incidence countries.

#### 2.1.2 Exclusion criteria

- Children with previous TB or LTBI.
- Tourist travel staying in hotels and/or resorts with scarce contact with the autochthonous population.
- Children with primary or secondary immunodeficiency due to treatment with corticosteroids, transplantation, treatment with anti-tumour necrosis factor, or chronic renal insufficiency.
- Children with congenital heart disease.
- Children with cystic fibrosis and other congenital pulmonary diseases.

### 2.2 Sample size

The sample size was calculated using the GRANMO v7.2, 2012 program for the study of paired proportions, accepting an alpha risk of 0.05 and a beta risk of 0.2 in a two-sided test. It was estimated that 492 subjects are necessary to obtain a statistically significant difference considering an initial LTBI proportion of 0% and a final proportion of 2%. A drop-out rate of 20% is anticipated.

### 2.3 Data collection

**Figure 2:**
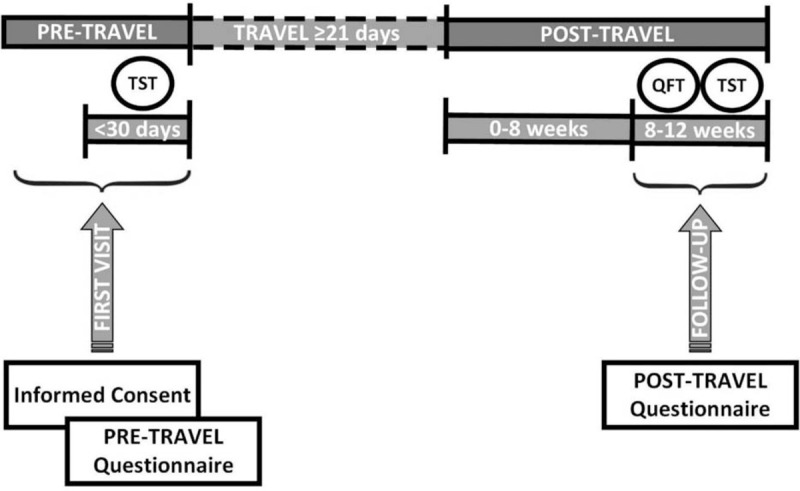
Timeline of study visits and procedures.

Baseline data collection: Eligible children will be offered to participate in the study during a programmed visit in a pre-travel health clinic or in a PCC. In the first visit, the parents or legal guardians will be interviewed to complete a questionnaire including socio-demographic, epidemiological, and clinical data. A tuberculin skin test (TST) will be performed and read 48 to 72 hours later. Figure 2 shows the study flowchart.

The TST will be made within 30 days prior to the trip. In BCG vaccinated children, TST will be performed and if the result is positive, a *QuantiFERON-Plus©* (QFT-Plus) test will be carried out to confirm the infection. Subsequently, if the QFT-Plus test is positive, the child will be excluded from the study and treated for LTBI.

Follow-up: A visit will be scheduled 8 to 12 weeks after returning from the trip. Here, a questionnaire with epidemiological and clinical data about the trip will be completed and a TST and QFT-Plus test will be simultaneously performed.

Children diagnosed with LTBI or TB, either at baseline or at follow-up, will receive treatment as per national guidelines, and relatives will be referred for contact tracing. Similarly, the investigators will verify the need for other preventive measures (malaria chemoprophylaxis, helminthiasis study, other immunizations, etc).

Data registration: The information collected in the questionnaires will be registered and managed in REDCap (Research Electronic Data Capture) tools hosted at Fundació de Docència I Recerca Mútua Terrassa. REDCap is a secure, web-based software platform designed to support data capture for research studies.^[21,22]^

### 2.4 Outcomes

#### 2.4.1 Primary outcome

The primary outcome is the diagnosis of LTBI and/or TB after travelling to a high TB incidence country in accordance with World Health Organization (WHO) guidelines.^[1]^ At the end of the study, patients will be classified as: “not infected; LTBI/TB disease; unknown/lost to follow-up”. Table 2 shows study variables and timing of data collection.

**Table 2:**
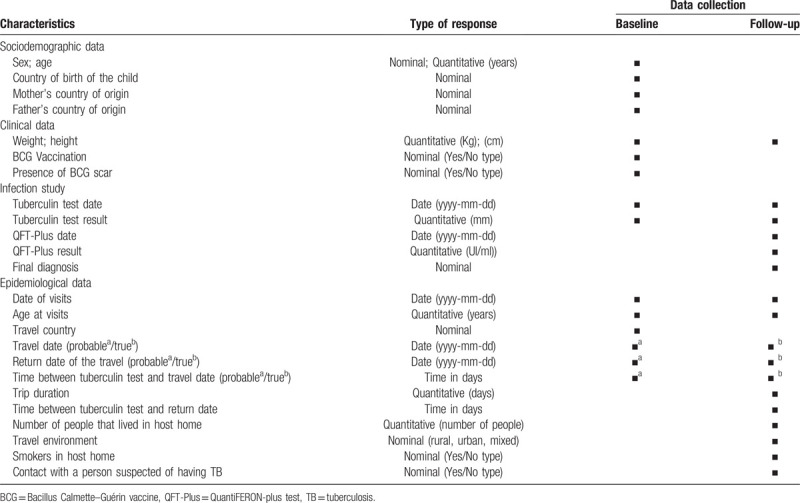
Study variables and timing of data collection.

#### 2.4.2 Secondary outcomes

1. Sociodemographic data: sex, age, country of birth, country of birth of mother and father.
2. Clinical data: weight and height (before and after travel), BCG vaccination, presence of BCG scar.
3. Epidemiological data: pre-travel visit date, travel date, post-travel visit date, return date of the travel, trip duration, travel country, number of people that lived in host home, travel environment (rural / urban / mixed), smokers in host home, contact with a person suspected of having TB.

### 2.5 TST and QFT-Plus test procedures

Tuberculin Skin Test (TST): TST will be performed by intradermal injection of 2 UT of PPD RT23 (Statens Serum Institut, Copenhagen, Denmark) and read after 48 to 72 hours. A positive test is defined as an induration greater than or equal to 10 mm according to recommendations from the *Agència de Salut Pública de Catalunya*^[23]^ and WHO guidelines.^[1]^

QuantiFERON-TB Plus (QFT-Plus) (Cellestis, Victoria, Australia; QIAGEN, Dusseldorf, Germany): We will draw four ml of blood per child and interpret the QFT-Plus test according to manufacturer's instructions. Values ≥ 0.35 IU/ml in either TB1 and/or TB2 antigen tubes will be considered positive.^[24]^ In the case of QFT-Plus values ≥0.2 and <0.35 (gray zone), the test will be repeated 4 to 8 weeks later. QFT-Plus can have a positive, negative or indeterminate result.

If TST and QFT-Plus results are discordant, the test with a negative result will be repeated 4 to 8 weeks later.

### 2.6 Statistical analysis

Qualitative variables will be described using frequency tables, and quantitative variables will be determined using median, mean and interquartile range (IQR). We will use paired proportions for comparisons. The incidence rate of LTBI and TB will be estimated per individual/month and person/year per country visited and by age group. To identify factors associated with presence of LTBI or TB, the Fisher exact test or Chi-square test and the Mann-Whitney test will be used for bivariate analyses, followed by analysis of logistic regression calculating odds ratios and 95% confidence intervals. A 5% type I error will be estimated. Data will be exported from the REDCap database to PASW Statistics 25 (SPSS Inc., Chicago, IL) for statistical analyses.

### 2.7 Ethics

This study protocol (version 2, 01/02/2016) has been approved by the Clinical Research Ethics Committee of the *Hospital Universitari Mútua Terrassa* on 24/02/2016 (code 02/16) and by the Clinical Research Ethics Committee of the *Fundació Institut Universitari per a la recerca a l’Atenció Primària de Salut Jordi Gol i Gurina* (IDIAPJGol) on 25/04/2016 (code P16/094), in agreement with the Declaration of Helsinki/Tokyo. All parents or legal guardians of the participants will receive oral and written information about the study and Informed Consent will be obtained. Any amendment to the study protocol will be submitted to the ethical committees for approval.

The REDCap© database will be exclusively used by the team of researchers. The data included in the database used for statistical analyses will be anonymized and identified with internal codes of the project to avoid identification by the investigative team to guarantee confidentiality.

The results of the study will be published in scientific journals and will be presented in national and international meetings. The results will be communicated to participants in a meeting and via local and national media, and will also be disseminated to the general population.

## 3 Discussion

This study aims to evaluate the risk of LTBI and TB in paediatric VFR travellers that live in Catalonia, a low incidence TB region.^[20]^ The study will be conducted in primary care centres, specialized travel clinics and hospital paediatric departments of the public health-care system. The joint role of primary care and infectious disease specialists is crucial in the implementation of strategies aimed at the eradication of TB in this population.^[25]^ To our knowledge, this is the first study to prospectively analyse collected data from VFR children.

The few studies published with adults to date suggest that in non-VFR travellers, the risk of infection is similar to the local annual risk of TB infection.^[26]^ In contrast, studies conducted with the immigrant population suggest that a significant percentage of TB cases are related to recent travel to the country of origin.^[27]^ In the case of children, a population group with higher susceptibility to develop TB after primary infection, a case-control study from California reported an increased risk of LTBI in young paediatric VFR travellers and children of families that hosted visitors from countries with high TB incidence.^[28]^

Awareness of travel-associated risk of disease in VFR remains low, which might explain the scarce use of pre-travel health-care services by the immigrant population.^[29]^ Moreover, the diversity of recommendations for different regions and countries demonstrates the limited information available on this topic.^[30,31]^ Knowledge about the effectiveness of LTBI screening or BCG vaccination strategies also remains poor.^[32]^ The implementation of strategies for the detection and treatment of LTBI cases should be prioritized to prevent new cases of TB in the paediatric and the adult population.^[33]^

Since state-level data are those usually published in official reports and regional incidence could vary significantly within the same country, a limitation of the study is the unavailability of data on regional incidence, which may hinder the assessment on the intensity of exposure to *M tuberculosis*.^[17]^ In addition, due to the specific immigration patterns of Catalonia, some countries of origin will be overrepresented in our sample.

The lack of gold standard for LTBI diagnosis responds to its paucibacillary nature, the quiescent state of *M tuberculosis* and the localization in the mediastinal lymph nodes.^[34]^ However, this is a universal limitation in all studies on this issue.

The low positive predictive value of the screening tests of LTBI in the general population constitutes another limitation. However, in low burden countries and among high risk groups such as immigrants and VFR travellers, tests may be useful for LTBI screening.^[35]^

Similarly, the TST and interferon-γ release assays have well described limitations, which can hinder the interpretation of results and the calculation of incidence rates, especially in the case of discordant results.^[36,37]^ Again, this is a universal limitation in all studies on this issue.

The study will implement and evaluate a new detection strategy and management of LTBI in VFR children in a country with a low incidence of TB. This strategy may prove useful to design public health policies in regions with low incidence of TB with significant numbers of VFR travellers.

## Acknowledgments

The authors thank the tuberculosis prevention and control program from the *Agència de Salut Pública de Catalunya* for study support.

## Author contributions

**Conceptualization:** Antoni Soriano-Arandes, Joan A. Caylà, Àngels Orcau, Antoni Noguera-Julian, Tomàs Maria Pérez-Porcuna.

**Data curation:** Alessandra Queiroga Gonçalves, Emma Padilla, Elisabet Solà-Segura, Neus Rius Gordillo, María Espiau, Mónica G. García-Lerín, Maria Àngels Rifà-Pujol, Jordi Gómez i Prat, Esperança Macia-Rieradevall, Andrea Martin-Nalda, Maria Eril-Rius, José Santos Santiago, Lídia Busquets-Poblet, Raisa Morales Martínez.

**Funding acquisition:** Antoni Soriano-Arandes, Tomàs Maria Pérez-Porcuna.

**Investigation:** Alessandra Queiroga Gonçalves, Emma Padilla, Elisabet Solà-Segura, Neus Rius Gordillo, María Espiau, Mónica G. García-Lerín, Maria Àngels Rifà-Pujol, Jordi Gómez i Prat, Esperança Macia-Rieradevall, Andrea Martin-Nalda, Maria Eril-Rius, José Santos Santiago, Lídia Busquets-Poblet, Raisa Morales Martínez, Tomàs Maria Pérez-Porcuna.

**Methodology:** Joan A. Caylà, Alessandra Queiroga Gonçalves, Àngels Orcau, Antoni Noguera-Julian.

**Project administration:** Antoni Soriano-Arandes, Tomàs Maria Pérez-Porcuna.

**Supervision:** Antoni Soriano-Arandes, Joan A. Caylà, Àngels Orcau, Antoni Noguera-Julian, Elisabet Solà-Segura, Neus Rius Gordillo, María Espiau, Mónica G. García-Lerín, Maria Àngels Rifà-Pujol, Jordi Gómez i Prat, Esperança Macia-Rieradevall, Andrea Martin-Nalda, Maria Eril-Rius, José Santos Santiago, Lídia Busquets-Poblet, Raisa Morales Martínez, Tomàs Maria Pérez-Porcuna.

**Visualization:** Antoni Soriano-Arandes, Joan A. Caylà, Àngels Orcau, Antoni Noguera-Julian, Tomàs Maria Pérez-Porcuna.

**Writing – original draft:** Antoni Soriano-Arandes, Joan A. Caylà, Alessandra Queiroga Gonçalves, Antoni Noguera-Julian, Tomàs Maria Pérez-Porcuna.

**Writing – review & editing:** Àngels Orcau, Emma Padilla, Elisabet Solà-Segura, Neus Rius Gordillo, María Espiau, Mónica G. García-Lerín, Maria Àngels Rifà-Pujol, Jordi Gómez i Prat, Esperança Macia-Rieradevall, Andrea Martin-Nalda, Maria Eril-Rius, José Santos Santiago, Lídia Busquets-Poblet, Raisa Morales Martínez.
